# Body mass index and overweight in relation to residence distance and population density: experience from the Northern Finland birth cohort 1966

**DOI:** 10.1186/1471-2458-13-938

**Published:** 2013-10-08

**Authors:** Simo Näyhä, Tiina Lankila, Arja Rautio, Markku Koiranen, Tuija H Tammelin, Anja Taanila, Jarmo Rusanen, Jaana Laitinen

**Affiliations:** 1 Institute of Health Sciences, University of Oulu, PO Box 5000, FI-90014 Oulu, Finland; 2 Center for Environmental and Respiratory Health Research, University of Oulu, PO Box 5000, FI-90014 Oulu, Finland; 3 Finnish Institute of Occupational Health, Aapistie 1, FI-90220 Oulu, Finland; 4 Department of Geography, University of Oulu, PO Box 8000, FI-90014, Finland; 5 Centre for Arctic Medicine, Thule Institute, University of Oulu, PO Box 7300, FI-90014 Oulu, Finland; 6 LIKES – Research Centre for Sport and Health Sciences, Viitaniementie 15a, FI-40720 Jyväskylä, Finland; 7 Primary Health Care Unit, Oulu University Hospital, PO Box 5000, FI-90014 Oulu, Finland

**Keywords:** Body mass index, Overweight, Medical geography, Urban/rural, Population density, Finland

## Abstract

**Background:**

The effect of urban sprawl on body weight in Finland is not well known. To provide more information, we examined whether body mass index (BMI) and the prevalence of overweight are associated with an individual’s distance to the local community centre and population density in his/her resident area.

**Methods:**

The sample consisted of 5363 men and women, members of the Northern Finland Birth Cohort 1966 (NFBC), who filled in a postal questionnaire and attended a medical checkup in 1997, at the age of 31 years. Body mass index (BMI; kg/m^2^) and the prevalence of overweight (BMI ≥ 25.0 kg/m^2^) were regressed on each subject’s road distance to the resident commune’s centre and on population density in the 1 km^2^ geographical grid in which he/she resided, using a generalized additive model. Adjustments were made for sex, marital status, occupational class, education, leisure-time and occupational physical activity, alcohol consumption and smoking.

**Results:**

The mean BMI among the subjects was 24.7 kg/m^2^, but it increased by increasing road distance (by 1.3 kg/m^2^ from 5–10 to 20–184 km) and by decreasing population density (by 1.7 kg/m^2^ from 1000–19,192 to 1–5 inhabitants/km^2^). The respective increases in overweight (overall prevalence 41%) were 13 per cent units for distance and 14 per cent units for population density. Adjusted regressions based on continuous explanatory variables showed an inverse L-shaped pattern with a mean BMI of 24.6 kg/m^2^ at distances shorter than 5 km and a rise of 2.6 kg/m^2^ at longer distances, and an increase of 2.5 kg/m^2^ from highest to lowest population density. The associations with road distance were stronger for women than men, while the sex difference in association with population density remained indeterminate.

**Conclusions:**

We conclude that young adults in Northern Finland who live far away from local centres or in the most sparsely populated areas are fatter than those who live close to local centres or in densely populated areas. The likely explanations include variations in everyday physical activity in different residential environments, although causality of the associations remains to be confirmed.

## Background

It is frequently observed that body weight is associated with the physical living environment. This is mostly described in conjunction with urban sprawl characterized by inadequate walkability of streets and roads, dependence on private cars and poor accessibility to well-equipped food stores [1-3], which would lead to a positive energy balance and increased body weight. Some authors report that the effects of urban sprawl on body mass index (BMI) in county-level comparisons can be as great as 1 kg/m^2^[3], which would markedly increase the prevalence of overweight and the risk of diabetes and cardiovascular diseases. The main underlying factor is considered to be everyday physical activity, which shows wide variations across countries. In the Netherlands, for example, 48% of trips are made by foot or bicycle, but only 10% in the USA [4]. From the public health point of view, the amount of physical activity undertaken in daily routines is more important than that used in sports and leisure-time physical exercise [3].

In Finland, studies on this topic are few, but they have revealed variations in body weight and obesity among administrative areas [5-7], community types [8,9] and 10-square-kilometre geographical grids [10]. The variations are attributed mostly to socioeconomic factors [10]. However, the physical community structures have greatly changed in Finland in recent decades due to in-migration to towns [11], and this may have affected the amount of physical activity needed in normal daily activities, with consequent changes in body weight. Therefore, any associations found between the physical community structures and body weight would be useful, especially as such associations are modifiable by community planning [12] and could provide tools for preventing obesity. This is particularly relevant in Finland, where the population is sparse and the distances are long.

The present study, based on a population-based cohort from northern Finland, tests the assumption that body weight and the prevalence of overweight depend on the physical structure of individuals’ residential environment defined by (1) road distance to the local community centre and (2) population density in the 1-square-kilometre geographical grid in which he or she resides. The former depicts the distance which the person would travel while doing his or her daily errands, and the latter not only population density itself but also the type of residence in the urban–rural scale [12]. The underlying rationale is that due to better connectivity, people walk and cycle more in densely populated than in sparsely populated areas [13].

## Methods

### The area studied

Finland is a subarctic country located between 60 and 70° N latitude and 20 and 31° E longitude. The population density in 1997 was 17 inhabitants per square kilometre, and the total land area is 338,145 km^2^, of which only 30% is inhabited. The country has experienced marked depopulation of the countryside in recent decades, with a consequent increase of the urban population and its density and a thinning of the rural population. This has caused sprawling of urban areas, with a simultaneous decrease of population density and an expansion of suburbs and build-up areas. The thinning of the rural population is expected to continue in the future [11].

**Figure 1:**
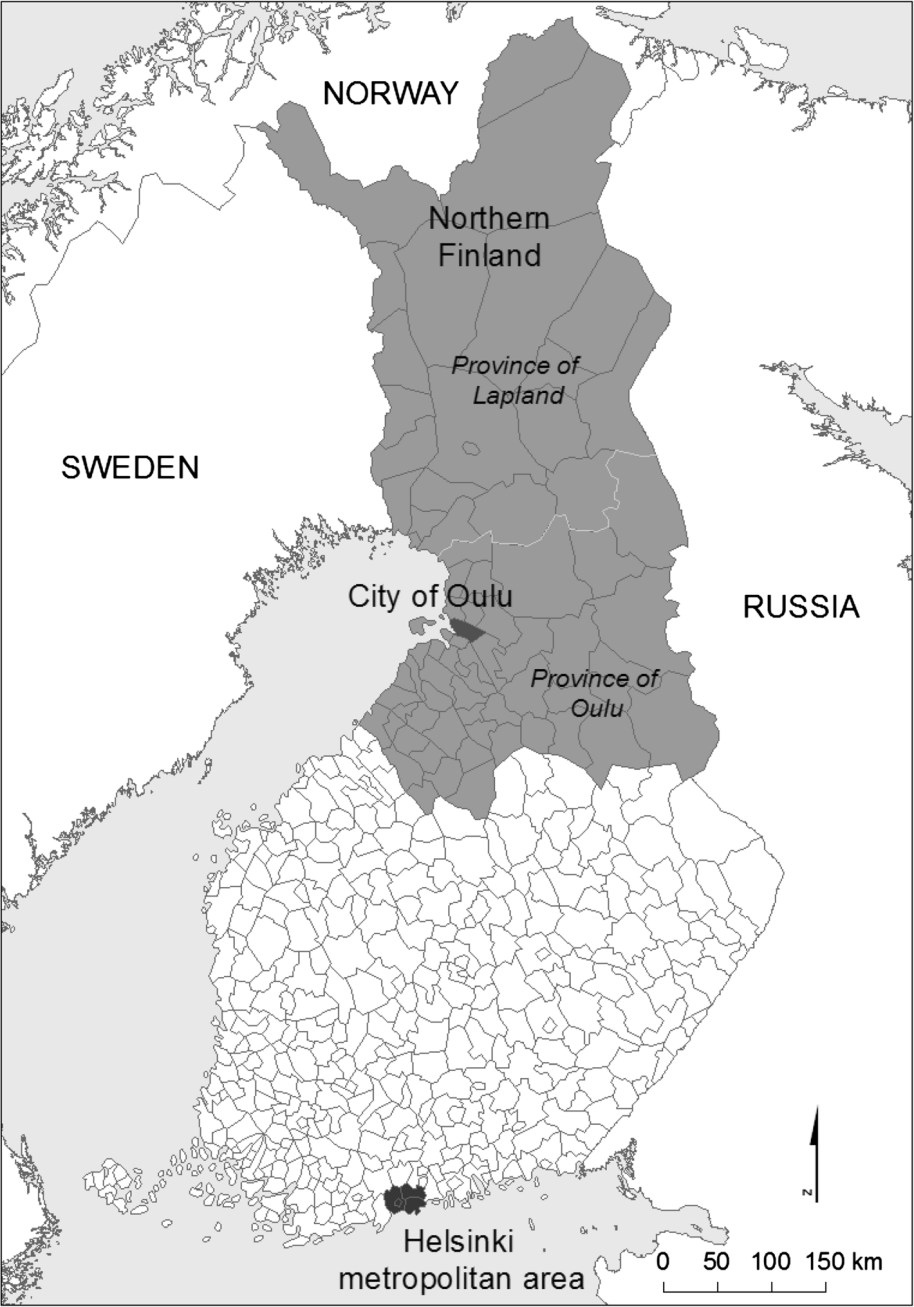
Northern Finland birth cohort study (1966): areas surveyed in 1997 shown as shaded.

In 1997, Finland was divided into 452 local government areas, called communes (Figure 1). Some of them (105 in 1997) are referred to as towns, while the rest are country communes, but all of them are equal in legal status. The communes differ widely with respect to land area (from 6 to 17,334 square kilometres), population (from 100 to 0.5 million) and population density (from 0.2 to 2760 inhabitants per km^2^). Especially in the far north, some settlements are located as far as 100 km or more from the local population centre. The study area (shown in grey in Figure 1) comprised three larger regions where the subjects lived in 1997: the city of Oulu, the rest of Northern Finland (provinces of Oulu and Lapland) and the Helsinki metropolitan area.

### The Northern Finland Birth Cohort (NFBC) data

The subjects were members of NFBC 1966, which consists of all individuals in the two northernmost provinces of Finland (Oulu and Lapland) whose mothers’ expected time of delivery was in 1966. The total number of births was 12,231 (12,058 live births), which covers 96.3% of all births in the area in 1966. The cohort have been followed up on since birth; the present analysis is based on a survey conducted in 1997, when the subjects were 31 years of age. A postal questionnaire was sent to all those 11,541 subjects who were still alive and whose addresses were known, and 8767 (76%) returned it. The respondents who lived in the provinces of Lapland and Oulu or in the Helsinki metropolitan area, altogether 6759 subjects, were invited to participate in a medical examination. Of those, 5713 (85%) attended, and 5690 filled in a separate questionnaire inquiring about working conditions, including a question on physical activity at work. The study was approved by the Ethical Committee of the Northern Ostrobothnia Hospital District.

### The road network data

To calculate each subject’s road distance to the local community centre, we used the Finnish road network data available in the Digiroad database, which is a national road and street database developed by the Finnish transport agency (see http://www.digiroad.fi/en_GB/). The database includes accurate geometry and length of road segments, which enables a calculation of actual distances between any two points along the road network. We used the earliest available version (2003) of the database. The road network in 2003 was essentially similar to that in 1997, as only a minimal number of new roads were built during that period; there were only renovations of the old road network [14,15].

### Coordinates of place of residence

Coordinates of the participants’ home address on 1 January 1997 were obtained from the Finnish Population Register Centre. Based on these coordinates, each cohort member living in the provinces of Oulu or Lapland or in the Helsinki metropolitan area and on whom the relevant health and work information was available, was attached to the 1-square-kilometre grid cell in which he/she resided using ArcGIS. A detailed description of how the grids were attached to the map is found elsewhere (http://www.stat.fi/meta/kas/yhtenaiskoordin_en.html). The record linkage failed for 327 subjects due to unavailability of the coordinates, errors in the datasets (incorrect coordinates in either data, coordinates that failed to match any inhabited grid or were on the boundary of two inhabited grids and therefore were ambiguous) and discrepant information on the resident commune in the cohort and grid data. This left 5363 subjects in the final study population.

### Outcomes

In the medical examination, body height and weight (to an accuracy of 0.1 cm and 0.1 kg, respectively) were measured and converted to body mass index (BMI; kg/m^2^). For a small proportion of the subjects (3.5%), body height and weight were based on self-reports in the questionnaire. Overweight was defined as BMI ≥ 25.0 kg/m^2^. BMI (a continuous variable) and overweight (yes/no) were used as outcomes.

### Explanatory variables

The shortest road distance between the subjects’ home and the midpoint of the densest populated 1-square-kilometre grid in his/her resident commune was used as the first explanatory variable and was calculated from the road network database. This was done using ArcGIS, by adding together the lengths of the individual road segments. The roads included regional and local main streets, collector streets, feeder streets and private streets, and in the costal archipelago, ferry connections between these streets. In the data analysis, distance was used as a continuous variable (in kilometres) but was also classified to arbitrary intervals (0–1.9 km, 2.0–4.9 km, 5.0–9.9 km, 10–19.9 km and 20–184 km) for descriptive purposes.

Population density in each 1-square-kilometre grid, obtained from Statistics Finland, was attached to each cohort member residing in this grid and was used as the second explanatory variable. Population density was analyzed as a continuous variable but was also classified to form residential area types: scattered settlements; rural areas proper; transitional zones; built-up areas & suburbs and high-rise centres having population densities of 1–5, 6–20, 21–100, 101–1000 and more than 1000 inhabitants per square kilometre, respectively [11].

### Potential confounding factors

Several suspected confounders were controlled for in the analysis. Firstly, we asked about the frequency of leisure-time physical activity (response options: once a month or less often; 2–3 times a month; once a week; 2–3 times a week; 4–6 times a week; daily) and the duration of physical activity at a time (not at all; less than 20 minutes; 20–39 minutes; 40–59 minutes; 1–1.5 hours; more than 1.5 hours), separately regarding light physical activity (no breathlessness or sweating ) and brisk physical activity (at least some breathlessness and sweating). The responses were converted to metabolic equivalents (MET) and MET hours per week. In the calculations, an intensity value of 3 METs was used for light physical activity and 5 METs for brisk physical activity [16]. Secondly, occupational physical activity was elicited by a question classifying the subjects as having light sedentary work, other sedentary work, light standing or moving work, medium heavy moving work, heavy manual work and very heavy manual work. In the analysis, the two lowest and two highest groups were merged (light sedentary/other sedentary work and heavy/very heavy manual work, respectively). The detailed questions have been described elsewhere [17,18].

Thirdly, the subjects were requested to indicate the frequency of their habitual consumption of rye or crisp bread, fresh vegetables, roots or salads, fruit or berries and sausages during the past six months (response options: less frequently than once a month or not at all; 1–2 times a month; once a week; a couple of times per week; almost every day; once a day or more often). An unhealthy diet was defined as one containing sausages daily or almost every day, and consumption of the rest of the food items a couple of times a week or less frequently [19].

Smoking was classified according to those smoking 5–7 days a week, 4 days a week or fewer and not smoking at all. The questionnaire also asked about the frequency of consumption of beer, wine and spirits during the last year, and the usual amount consumed per drinking occasion. The daily amount of alcohol consumed was calculated using the following alcohol contents (vol %): beer: 4.8; light wine: 5.0; table wine: 14.5; and spirits: 37.0. The subjects were classified into quartiles of alcohol consumption (grams per day). The method has been validated against 7-day food records [19].

In addition to sex and marital status, demographic factors allowed for in the analysis were based on self-reports and included socioeconomic group (entrepreneurs; higher administrative employees; lower administrative employees; blue collar workers; other) [20], occupational class (office work; industry; agriculture/forestry; health & social work; business) [21] and education (university; college or polytechnic; vocational school or course; no vocational school).

### Statistical analysis

We first drew the histograms of residence distance and population density which we smoothed by Gaussian kernel density function using smoothing windows of 0.5 km and 150 inhabitants/km^2^, respectively. Then BMI and the prevalence of overweight were regressed on logged distance and population density using a generalized additive model in which smoothing was achieved by cubic splines with 4 degrees of freedom. We used the Gaussian error distribution for BMI and binomial for overweight, and the identity link function in both cases. The results were expressed as smoothed predictions of BMI (kg/m^2^) and the prevalence of overweight together with their 95% confidence bands. The method has the advantage of retaining the continuity of the explanatory variables without assuming any regular shape of the relationship. Confounders were added to the models depending on whether they caused any marked change in the smoothed predictions. The intra-class correlations of BMI and overweight within the resident communes were close to zero (< 0.01). The calculations were performed using the R software, release 2.15.0 [22].

## Results

### Descriptive data

**Figure 2:**
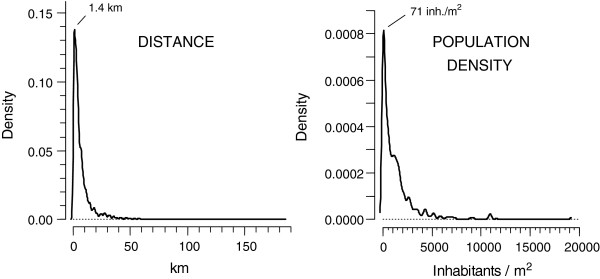
**Distribution of subjects according to residence distance and population density in the resident grid.** Distribution of subjects according to distance to the midpoint of resident commune’s densest grid and according to population density of the resident grid. Smoothed by Gaussian kernel density function with smoothing windows of 0.5 km (distance) and 150 inhabitants per km^2^ (population density). The modal values of each distribution are also indicated.

Figure 1 shows the study area broken down by communes and larger regions, and (Additional file 1: Figure S1) depicts the roads (blue lines) used to calculate each individual’s shortest distance to his or her resident commune’s centre. The cohort members were heavily concentrated at short distances and densely populated grids (Figure 2). Thus 92% of the subjects lived at distances shorter than 20 km, the most typical distance being 1.4 km, and most of them (76%) lived in grids having more than 100 inhabitants per square km, most typically in grids with 71 inhabitants/km^2^.

The majority of the subjects (86%) still lived in the city of Oulu or elsewhere in northern Finland, while 15% had moved to the Helsinki metropolitan area. Most subjects (93%) worked in service occupations, the health sector, business or industry; 19% were higher administrative employees; 10% had university education and 20% were engaged in heavy physical work; 11% had unhealthy diet, and 27% smoked on at least five days a week (details are shown in Table 1).

**Table 1:** Distribution of the subjects according to major demographic, work-related and lifestyle factors, road distance from resident commune’s centre and population density of the subject’s resident grid

**Demographic, work-related and lifestyle factors**	**No. (%) of subjects**		**Road distance (km)**		**Population density (inhabitants/km**^**2**^**)**	
			**P**_**50**_	**P**_**95**_	**P**_**50**_	**P**_**95**_
**Sex**						
Men	2550	(47.5)	4.2	28.1	678	4506
Women	2813	(52.5)	4.1	26.0	719	5181
Total	5363	(100.0)	4.1	27.3	712	4986
**Marital status**						
Married/cohabiting	3789	(72.4)	4.3	25.4	597	4188
Other	1444	(27.6)	3.6	31.4	1037	6823
Total	5233	(100.0)	4.1	27.3	713	4986
**Region of residence**						
City of Oulu	1121	(21.0)	4.9	9.8	1537	4325
Rest of northern Finland	3447	(64.5)	3.3	32.6	306	2267
Helsinki metropolitan area	774	(14.5)	7.3	17.6	3093	10899
Total	5342	(100.0)	4.1	27.3	712	4986
**Occupational class**						
Office work^a^	2049	(42.9)	4.1	21.6	949	5696
Industry^b^	1193	(25.0)	3.8	28.7	538	4325
Agriculture^c^	329	(6.9)	11.2	50.7	27	1076
Health/social work	806	(16.9)	3.8	19.4	881	4839
Business	404	(8.5)	3.7	19.9	933	4363
Total	4781	(100.0)	4.2	29.4	525	4325
**Socio-economic group**						
Entrepreneurs	357	(6.9)	7.4	42.2	46	2816
Higher administrative	988	(19.0)	4.2	15.9	1361	6566
Lower administrative	1877	(36.1)	3.8	21.2	799	5176
Blue collar workers	1702	(32.7)	4.1	31.2	507	3765
Other^d^	274	(5.3)	3.7	29.0	960	5181
Total	5198	(100.0)	4.1	27.2	716	5075
**Education**						
University	546	(10.0)	4.2	14.4	1515	7178
College/polytechnic	1835	(34.9)	3.9	19.7	911	5181
Vocational school/course	1872	(35.6)	4.4	31.9	435	3754
No vocational school	1026	(19.5)	4.0	27.7	614	4325
Total	5259	(100.0)	4.1	27.3	710	4986
**Occupational physical activity**						
Sedentary work	1637	(41.3)	4.3	18.2	1102	5884
Light standing or moving work	670	(16.9)	3.6	20.0	911	5181
Medium heavy moving work	858	(21.6)	4.2	28.1	539	4663
Heavy/very heavy manual work	799	(20.2)	4.8	31.4	368	4043
Total	3964	(100.0)	4.3	24.5	747	5181
**Leisure-time physical activity**^**e **^**(quintiles, MET-hours per week)**						
0–2.7	1020	(19.7)	4.4	28.0	506	4362
2.8–7.7	1046	(20.2)	4.5	26.9	610	4736
7.8–14.1	1046	(20.2)	4.0	24.4	845	5181
14.2–25.0	1030	(19.9)	3.9	23.6	942	5181
25.1–84.0	1034	(20.0)	3.7	28.9	713	5075
Total	5176	(100.0)	4.1	27.1	717	5075
**Diet**^**f**^						
Healthy	4758	(88.7)	4.1	27.0	717	5075
Unhealthy	605	(11.3)	4.0	29.1	640	4329
Total	5363	(100.0)	4.1	27.3	712	4986
**Alcohol consumption (quartiles, grams per day)**						
0.0–1.0	1288	(24.9)	4.3	32.6	392	3625
1.1–4.0	1282	(24.8)	4.2	25.7	637	4325
4.1–10.4	1305	(25.2)	4.1	24.2	884	4964
10.5–531	1298	(25.1)	3.8	22.3	971	6628
Total	5173	(100.0)	4.1	27.3	710	4986
**Smoking**						
On 5–7 days/week	1443	(27.4)	3.6	24.4	713	4325
On 4 days/week at most^g^	690	(13.1)	4.2	25.9	926	5619
Not at all	3128	(59.5)	4.3	27.8	640	5181
Total	5261	(100.0)	4.1	27.3	713	4986
**All**	5363		4.1	27.3	712	4986

^a^Office work, administration, services.

^b^Transport, construction, factory work.

^c^Farming, forestry, stock raising.

^d^Students, pensioners, unemployed or socioeconomic group unknown.

^e^See text for explanation.

^f^Diet including daily or almost daily consumption of sausages, and consumption of rye or crisp read, fresh vegetables, roots or salads, fruits or berries twice a week or less often, one point being assigned on each of these counts, so that a sum of 4 to 5 points indicated an unhealthy diet and 3 or fewer a healthy diet.

^g^Smokes on 1–4 days/week or only occasionally.

The median distance from home to the resident commune’s centre was 4.1 km, but it varied among subgroups (Table 1). Thus the subjects living in Oulu or elsewhere in northern Finland resided closer (median distance 4.9 km and 3.3 km, respectively) than those living in the Helsinki metropolitan area (7.3 km). The longest distances in Oulu and Helsinki were only 25 km and 26 km, respectively, but could reach 184 km in the countryside. People engaged in agriculture lived farther (median distance 11.2 km) than those engaged in other occupations (3.7–4.1 km), and entrepreneurs lived farther (7.4 km) than people belonging to other socioeconomic groups (3.7–4.2 km). Table 1 also shows variations in residence distance depending on marital status, education and lifestyle factors, but these were minor or inconsistent.

The median population density in the subjects’ resident grid was 712 inhabitants/km^2^. The subjects living in Helsinki, those who were not married, those who were engaged in occupations other than agriculture, those who were higher administrative employees and those who had university education lived in relatively dense grids (Table 1). The median population density increased with increasing leisure-time physical activity and decreasing occupational physical activity. Population density also increased with growing alcohol consumption, and smokers and subjects who had a healthy diet lived in relatively dense grids.

### Crude associations of BMI and overweight with residence distance, population density and potential confounders

BMI averaged 24.7 kg/m^2^, and 41% of the subjects were overweight (BMI ≥ 25 kg/m^2^). The mean BMI showed a curved pattern depending on residence distance, the values being 0.7–1.3 kg/m^2^ higher at distances of at least 20 km compared with shorter distances, the corresponding prevalence difference being 6–13 per cent units (Table 2). The mean BMI was lowest in high-rise centres (at least 1000 inhabitants/km^2^) and increased consistently by 1.7 kg/m^2^ to scattered settlement areas (1–5 inhabitants/km^2^), a similar trend being seen in overweight (the respective difference being 14 per cent units).

**Table 2:** Crude associations of body mass index (BMI) and overweight with road distance, residential area type and demographic and lifestyle factors

	**BMI (kg/m**^**2**^**)**		**No. (%) overweight (BMI ≥ 25)**	**No. of subjects**
	**Mean**	**SD**^**a**^		
**Road distance to commune’s centre (km)**				
0.0–1.9	24.71	4.25	549 (41.2)	1334
2.0–4.9	24.50	4.20	661 (38.3)	1727
5.0–9.9	24.45	3.97	449 (38.0)	1183
10.0–19.9	25.10	4.51	295 (45.2)	652
20.0–184.0	25.76	4.79	206 (51.4)	401
**Residential area type (population density, inhabitants per km**^**2**^**)**				
Scattered settlements (1–5)	26.07	5.34	74 (52.1)	142
Rural areas proper (6–20)	25.35	4.46	200 (45.5)	440
Transition zones (21–100)	25.22	4.80	292 (43.8)	667
Built-up areas & suburbs (101–1000)	24.65	4.10	734 (41.1)	1785
High-rise centres (1000–19192)	24.40	4.06	860 (38.0)	2263
**Sex**				
Men	25.25	3.62	1246 (49.6)	2513
Women	24.22	4.72	914 (32.8)	2784
**Marital status**				
Married/cohabiting	24.73	4.10	1550 (41.0)	3779
Other	24.68	4.70	579 (40.2)	1441
**Region of residence**				
City of Oulu	24.52	4.00	439 (39.6)	1109
Rest of northern Finland	24.94	4.41	1458 (42.7)	3416
Helsinki metropolitan area	23.99	3.88	263 (34.1)	772
**Occupational class**				
Office work	24.40	4.32	749 (36.6)	2044
Industry	25.21	3.98	585 (49.2)	1190
Agriculture	25.25	4.45	146 (44.6)	327
Health/social work	24.37	4.44	283 (35.2)	805
Business	24.64	3.99	165 (40.8)	404
**Socio-economic group**				
Entrepreneurs	25.32	4.26	164 (45.9)	357
Higher administrative	24.11	3.57	348 (35.3)	985
Lower administrative	24.49	4.40	697 (37.2)	1875
Blue collar workers	25.19	4.35	797 (47.0)	1696
Other	24.57	4.54	105 (38.6)	272
**Education**				
University	23.84	3.55	160 (30.6)	523
College/polytechnic	24.41	3.98	704 (38.4)	1831
Vocational school/course	25.14	4.34	853 (45.6)	1869
No vocational school	24.87	4.79	419 (41.0)	1023
**Occupational physical activity**				
Sedentary work	24.54	4.10	639 (39.4)	1620
Light standing or moving work	24.30	3.97	242 (36.4)	664
Medium heavy moving work	24.65	3.87	350 (41.2)	850
Heavy/very heavy manual work	25.17	4.19	363 (45.8)	793
**Leisure-time physical activity (quintiles, MET-hours per week)**				
0–2.7	25.26	4.72	482 (47.3)	1019
2.8–7.7	24.92	4.54	441 (42.3)	1042
7.8–14.1	24.50	4.06	400 (38.3)	1044
14.2–25.0	24.39	3.92	385 (37.5)	1027
25.1–84.0	24.37	3.82	390 (37.8)	1031
**Diet**				
Healthy	24.66	4.26	1876 (40.0)	4694
Unhealthy	25.14	4.45	284 (47.1)	603
**Alcohol consumption (quartiles, grams per day)**				
0.0–1.0	24.81	5.05	499 (38.9)	1284
1.1–4.0	24.48	4.15	486 (38.0)	1278
4.1–10.4	24.40	3.86	491 (37.7)	1302
10.5–531	25.17	3.83	628 (48.4)	1297
**Smoking**				
On 5–7 days/week	24.85	4.50	613 (42.5)	1441
On 4 days/week at most	25.03	4.12	305 (44.2)	690
Not at all	24.57	4.17	1217 (39.0)	3118
**All**	24.71	4.26	2160 (40.8)	5297

^a^ standard deviation.

Table 2 also shows higher BMI and higher prevalence of overweight in men than women, in subjects living in northern Finland versus those living in Helsinki, in entrepreneurs compared with other socioeconomic groups and in people having lower education versus higher education. The subjects working in agriculture and industry were fattest, and those in the health sector were leanest. The BMI and the prevalence of overweight decreased with increasing leisure-time physical activity, while the subjects doing heavy physical work had the highest BMI and those doing light physical work the lowest. Unhealthy diet was associated with a slightly higher BMI and more overweight than healthy diet, and smokers were fatter than non-smokers. Subjects in the highest alcohol consumption quartile had higher BMI and more overweight compared with those in other quartiles.

### Analyses allowing for confounders and curvilinear associations

**Figure 3:**
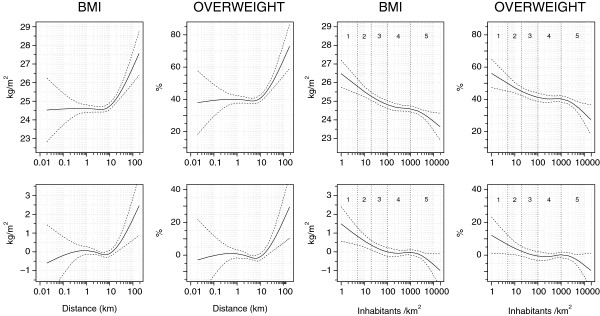
**BMI (kg/m**^**2**^**) and overweight (BMI ≥ 25 kg/m**^**2**^**) according to residence distance and population density.** Body mass index (BMI; kg/m^2^) and percentage of overweight (BMI ≥ 25 kg/m^2^) in relation to individual’s road distance (km) to the resident commune’s densest grid, and on population density of the resident grid (inhabitants/km^2^). Continuous line indicates the regression-based estimate for BMI and the prevalence of overweight, smoothed by a cubic spline with 4 degrees of freedom (95% confidence bands shown by dashed lines). Residential area types are marked by Arabic numerals: 1 scattered settlements; 2 rural areas proper; 3 transition zones; 4 built-up areas & suburbs; 5 high-rise centres. *Upper row:* crude BMI and prevalence of overweight. *Lower row:* regression-based gradients compared with the baseline, adjusted for sex, marital status, occupational class, education, leisure-time and occupational physical activity, alcohol consumption and smoking.

The unadjusted regressions using road distance as a continuous explanatory variable (Figure 3) showed an inverse L-shaped pattern with a relatively low BMI (24.6 kg/m^2^) at distances shorter than 5 km and a smooth rise of 3.0 kg/m^2^ at distances longer than that. The pattern was similar for overweight, the prevalence increasing by 35 per cent units beyond the distance of approximately 5 km. In further analyses adjusting for sex, marital status, occupational class, education, leisure-time and occupational physical activity, alcohol consumption and smoking, BMI and overweight still increased beyond the distance of 5 km, the increases being slightly smaller (2.6 kg/m^2^ and 30 per cent units, respectively). An additional allowance for socio-economic group and diet caused no appreciable changes in the smoothed curves.

A breakdown by sex in (Additional file 2: Figure S2) indicated greater distance-related increases in BMI beyond 5 km among women than men, in both unadjusted (women 3.5, men 1.9 kg/m^2^) and adjusted (women 3.0, men 2.1 kg/m^2^) analyses, and a corresponding sex difference in overweight (unadjusted increase 37 versus 20 per cent units in women and men, respectively; adjusted increase 40 versus 18 per cent units, respectively). However, the wider confidence intervals and less precise regression estimates left the associations more equivocal than in the unstratified analysis.

BMI and overweight also increased with decreasing population density, even though the relation was less curvilinear than that for residence distance (Figure 3). The unadjusted model-estimated increase over the whole population density scale was 2.8 kg/m^2^ for BMI and 28 per cent units for overweight, and these diminished to respectively 2.5 kg/m^2^ and 20 per cent units when allowance was made for the confounders mentioned above. The sex differences were less consistent than those regarding distance-related sex differences (Additional file 2: Figure S2).

## Discussion

We found that among young Finnish adults, BMI and the prevalence of overweight increased with increasing road distance to the local commune centre, but only at distances longer than approximately 5 kilometres, and they increased from densely to sparsely populated areas. This is the first attempt in Finland to explain the geographical variation in body weight [6-10] in terms of residence distance and grid-based population density. The variations of BMI and overweight were large, and in case the associations can be interpreted causally, the findings provide opportunities for prevention of overweight by health-based planning of the built environment.

The strength of our study is the large population-based cohort in well-defined geographical areas of Finland, where the distances and population density vary widely. The geographical grids on which the information on population density was based were smaller than those used previously [10] and were small enough to reflect influences of individuals’ close environment. The response rate was satisfactory, and any marked selection at different phases of the study is unlikely. We were able to use road distance as an explanatory factor, which is likely to serve as a reasonable estimate for the length of actual distances people go for their daily trips—although we did no individual measurements nor did we measure the duration of the trips.

One limitation of the study is that we had no information on the actual length of work travels or on the mode of travelling to work. Therefore, we cannot differentiate between the effects of leisure time physical activity and those related to commuting. Some confounding may have been caused by higher unemployment rates and consequently less work-related physical activity in sparsely populated areas, which was not specifically controlled for. We did control for socioeconomic group, in which unemployed persons are included in the class “other”, but this did not markedly change the parameter estimates and this factor was not included in the final model.

The questions on leisure-time and occupational physical activity have been previously used and have adequate face validity [17,18]. We similarly assume that the composite variable used to measure the healthiness of diet is valid because it predicts abdominal obesity [19], even though calorie intake was not measured. We cannot rule out a possibility of residual confounding, but we believe that our findings serve as reasonable estimates for how much residence distance and population density may affect body weight.

Geographical studies of BMI and obesity conducted in Finland have found variations between localities [5] and larger areas [6] and a lower BMI in the capital area of Helsinki than in other areas [7]. The sole grid-based analysis of body weight conducted in this country observed a lower prevalence of obesity in cities compared with other areas [10]. Investigations performed elsewhere have either found [23] or have not found [24] more obesity in rural than in urban areas. The lower BMI in urban areas has been linked with higher socioeconomic status [9] and higher population density [2,25-27], and is interpreted in terms of proximity of daily destinations accessible by walking or bicycle and the consequently greater physical activity. We used road distance as an operational measure for actual distances which people travel in everyday life, and population density to indicate the subject’s community type in urban–rural scale. By a reasonable assumption, both measures are related to the intensity of physical activity and the accessibility of services and amenities, the former on account of travel lengths and mode of moving, the latter through better connectivity with daily destinations.

We observed higher body weight among people living in remote and sparsely populated areas. The association was attributable partly to social, demographic and lifestyle factors including leisure-time and occupational physical activity. As the associations persisted after adjustments, true effects of distance and population density are a possibility. The cross-sectional study design and the possibility of unidentified confounders prevent us from drawing causal conclusions. We also acknowledge a possibility of migration-related bias. A Finnish study concluded that heavier adolescents who reside in the countryside may be less likely to move to towns than normal weight adolescents [9], but we do not know whether this is true for adults.

Substantive evidence from elsewhere favours the view that individuals’ physical environment modifies the amount of physical activity in daily life. The relevant issues include how far away the daily destinations are located and whether the distances are travelled by foot, bicycle, public transport or private car. More overweight has been reported among people who live far from large supermarkets [28] or recreational facilities [29] or who have poor access to them [30,31], those who live far from bike paths [32] or have no shops or paths within walking distance [30] and those who have few non-residential destinations [29]. Proximity of employment establishments, grocery stores and business centres is believed to be linked to lower body weight [33].

Less overweight has been observed in individuals who have an easy access to healthy food stores [34] or supermarkets [35] or have healthy grocery or multiple food options in their neighbourhood [12], while people living close to fast-food restaurants are fatter [36]. Factors associated with low BMI and less obesity also include easy access to green areas [37,38] or living in neighbourhoods with much vegetation [28]. Increased walkability of streets would decrease the risk of overweight [39], each kilometer walked per day decreasing the likelihood of obesity by 5% and each hour spent in a car per day increasing the likelihood of obesity by 6% [40]. People are leaner in areas in which a higher percentage of the population walks to work [12], while those using a car for work travels or trips to grocery stores are fatter [41,42].

## Conclusions

Even in sparsely populated Finland, urban centres have sprawled in recent decades as a consequence of migration of the country population to towns [11], but the public health consequences of this with respect to body weight are not well recognized nor taken into account in the national health policy. However, the high-level health and population records in Finland provide excellent opportunities to foresee and control the adverse effects of urban sprawl and rural depopulation. Our results suggest that approximately 40% of young adults live in areas that may adversely affect their body weight because their place of residence is located too far away from local centres or is too sparsely inhabited. We assume that variations in everyday physical activity in different areas explain the findings, but individual-based measurements of daily activity are needed to strengthen this causal argument. The prevalence of overweight attributable to geographical location represents a significant and removable fraction of the public health burden and calls for more studies on the effects of community planning on health [4]. Even though causality needs to be confirmed for the associations reported here, the findings emphasize that community planning is a health issue that should be closely examined not only by city planners, but also by public health scientists.

## Competing interests

The authors declare that they have no competing interests.

## Authors’ contributions

The study was conceived by SN, TL and JR. The data analysis was done by SN and MK, and the manuscript was drafted by SN and TL and revised and finalized by SN, TL, JR, JL, AR, THT and AT. All authors have read and approved the final manuscript. SN, JR and AT are the guarantors of the study.

## Pre-publication history

The pre-publication history for this paper can be accessed here:

http://www.biomedcentral.com/1471-2458/13/938/prepub

## Supplementary Material

Additional file 1: Figure S1 Detailed map of the area studied. Detailed map of the area studied, showing the entire road network (marked in grey) and the roads used to calculate each individual’s travel distance to the resident commune’s centre (blue). Click here for file

Additional file 2: Figure S2 BMI (kg/m^2^) and overweight (BMI ≥ 25 kg/m^2^) according to residence distance and population density, by sex. Body mass index (BMI; kg/m^2^) and percentage of overweight (BMI ≥ 25 kg/m^2^) in relation to individual’s road distance (km) to the resident commune’s densest grid, and on population density of the resident grid (inhabitants/km^2^), separately for men and women. Continuous line indicates the regression-based estimate for BMI and the prevalence of overweight, smoothed by a cubic spline with 4 degrees of freedom (95% confidence bands shown by dashed lines). Residential area types are marked by Arabic numerals: 1 scattered settlements; 2 rural areas proper; 3 transition zones; 4 built-up areas & suburbs; 5 high-rise centres. *Upper rows:* crude BMI and prevalence of overweight. *Lower rows:* regression-based gradients compared with the baseline, adjusted for marital status, occupational class, education, leisure-time and occupational physical activity, alcohol consumption and smoking. Click here for file

## References

[B1] Garden FL Jalaludin BB Impact of urban sprawl on overweight, obesity, and physical activity in Sydney, Australia J Urban Health 2008 86 19 30 19052877 10.1007/s11524-008-9332-5 PMC2629517

[B2] Lovasi GS Neckerman KM Quinn JW Weiss CC Rundle A Effect of individual or neighborhood disadvantage on the association between neighborhood walkability and body mass index Am J Public Health 2009 99 279 284 10.2105/AJPH.2008.138230 19059849 PMC2622783

[B3] McCann BA Ewing R Measuring the health effects of sprawl. A national analysis of physical activity, obesity and chronic disease. Smart Growth America. Surface Transportation Policy Project 2003 Washington DC http://smartgrowth.umd.edu/assets/ewingmccann_2003.pdf

[B4] Frumkin H Urban sprawl and public health Public Health Rep 2002 117 201 217 12432132 10.1093/phr/117.3.201 PMC1497432

[B5] Heliövaara M Aromaa A Height, weight and obesity of Finnish adults 1980 Helsinki, Finland: Publications of the Social Insurance Institution, Finland, ML:19 Finnish, with English summary

[B6] Näyhä S Hassi J Näyhä S, Hassi J Life style, work and health of Finnish reindeer herders Life style, work and health of Finnish reindeer herders. Helsinki: The Finnish Social Insurance Institution 1993 Finland: Publications of the Social Insurance Institution 217 237 ML:127 ]

[B7] Pietinen P Vartiainen E Männistö S Trends in body mass index and obesity among adults in Finland from 1972 to 1992 Int J Obes 1996 20 114 120 8646247

[B8] Fogelholm M Valve R Absetz P Heinonen H Uutela A Patja K Karisto A Konttinen R Mäkelä T Nissinen A Jallinoja P Nummela O Talja M Rural–urban differences in health and health behaviour: a baseline description of a community health-promotion programme for the elderly Scand J Public Health 2006 34 632 640 10.1080/14034940600616039 17132597

[B9] Jokela M Kivimäki M Elovainio M Viikari J Raitakari OT Keltikangas-Järvinen L Urban/rural differences in body weight. Evidence for social selection and causation hypothesis in Finland Soc Sci Med 2009 68 867 875 10.1016/j.socscimed.2008.12.022 19147263

[B10] Lahti-Koski M Taskinen O Similä M Männistö S Laatikainen T Knekt P Valsta LM Mapping geographical variation in obesity in Finland Eur J Public Health 2008 18 637 643 10.1093/eurpub/ckn089 18854358

[B11] Rusanen J Muilu T Colpaert A Naukkarinen A Georeferenced data as a tool for monitoring the concentration of population in Finland in 1970–1998 Fennia, Int J Geography 2003 181 129 144

[B12] Zick CD Smith KR Fan JX Brown BB Yamada I Kowaleski-Jones L Running to the store? The relationship between neighborhood environments and the risk of obesity Soc Sci Med 2009 69 1493 1500 10.1016/j.socscimed.2009.08.032 19766372 PMC2791711

[B13] Saelens BE Sallis JF Frank LD Environmental correlates of walking and cycling: Findings from the transportation, urban design, and planning literatures Ann Behav Med 2003 25 80 91 10.1207/S15324796ABM2502_03 12704009

[B14] Finnish road statisticshttp://www2.liikennevirasto.fi/julkaisut/pdf3/lti_2011-06_tietilasto_2010_web.pdf

[B15] Uimonen S Measuring the highway capital in Finland 1900–2009. Tampere Economic Working Papers Net Series 81 2010 Tampere, Finland http://urn.fi/urn:isbn:978-951-44-8226-7

[B16] Kaakinen M Läärä E Pouta A Hartikainen AL Laitinen J Tammelin TH Herzig KH Sovio U Bennet AJ Peltonen L McCarthy MI Elliot P DeStavola B Järvelin MR Life course analysis of a fat mass and obesity-associated (FTO) gene Variant and body mass index in the Northern Finland birth cohort 1966 using structural equation modelling Am J Epidemiol 2010 172 653 665 10.1093/aje/kwq178 20702506 PMC2938267

[B17] Hu G Qiao Q Silventoinen K Eriksson JG Jousilahti P Lindström J Valle TT Nissinen A Tuomilehto J Occupational, commuting, and leisure-time physical activity in relation to risk for type 2 diabetes in middle-aged Finnish men and women Diabetologia 2003 46 322 329 12687329 10.1007/s00125-003-1031-x

[B18] Tammelin T Näyhä S Rintamäki H Zitting P Occupational physical activity is related to physical fitness in young workers Med Sci Sports Exer 2002 34 158 165 10.1097/00005768-200201000-00024 11782662

[B19] Laitinen J Pietiläinen K Wadsworth M Sovio U Järvelin MR Predictors of abdominal obesity among 31-y-old men and women born in Northern Finland in 1966 Eur J Clin Nutr 2004 58 180 190 10.1038/sj.ejcn.1601765 14679384

[B20] Statistics Finland Sosioekonomisen aseman luokitus 1989 1989 Helsinki, Finland: Käsikirjoja 17 Classification of social status 1989: in Finnish

[B21] Statistics Finland Ammattiluokitus 1987 1987 Helsinki, Finland: Käsikirjoja 14 Classification of occupations; in Finnish

[B22] R Development Core Team: R A language and environment for statistical computing. Release 2.15.0 2012 Vienna, Austria: R Foundation for Statistical Computing http://www.R-project.org

[B23] Reeder BA Chen Y Mcdonald SM Angel A Sweet L Regional and urban–rural differences in obesity in Canada. Canadian Heart Health Survey Group Can Med Assoc J 1997 57 Suppl 1 S10 S16 9220949

[B24] Peytremann-Bridevaux I Faeh D Santos-Eggimann B Prevalence of overweight and obesity in rural and urban settings in 10 European countries Prev Med 2007 44 442 446 10.1016/j.ypmed.2006.11.011 17258803

[B25] Lee IM Ewing R Sesso HD The built environment and physical activity levels: the Harvard Alumni Health Study Am J Prev Med 2009 37 293 298 10.1016/j.amepre.2009.06.007 19765500 PMC2749578

[B26] Rundle A Roux AV Free LM Miller D Neckerman KM Weiss CC The urban built environment and obesity in New York City: a multilevel analysis Am J Health Promot 2007 21 4 Suppl 326 334 17465178 10.4278/0890-1171-21.4s.326

[B27] Zhao Z Kaestner R Effects of urban sprawl on obesity J Health Econ 2010 29 779 787 10.1016/j.jhealeco.2010.07.006 20832131

[B28] Liu GC Wilson JS Qi R Ying J Green neighborhoods, food retail and childhood overweight: differences by population density Am J Health Promot 2007 21 4 Suppl 317 325 17465177 10.4278/0890-1171-21.4s.317

[B29] Boehmer TK Lovegreen SL Haire-Joshu D Brownson RC What constitutes an obesogenic environment in rural communities? Am J Health Promot 2006 20 411 421 10.4278/0890-1171-20.6.411 16871821

[B30] Giles-Corti B Macintyre S Clarkson JP Pikora T Donovan RJ Environmental and lifestyle factors associated with overweight and obesity in Perth, Australia Am J Health Promot 2003 18 93 102 10.4278/0890-1171-18.1.93 13677967

[B31] Gordon-Larsen P Nelson MC Page P Popkin BM Inequality in the built environment underlies key health disparities in physical activity and obesity Pediatrics 2006 117 417 424 10.1542/peds.2005-0058 16452361

[B32] Petrella RJ Kennedy E Overend TJ Geographic determinants of healthy lifestyle change in a community-based exercise prescription delivered in family practice Environ Health Insights 2008 1 51 62 21572848 10.4137/EHI.S820 PMC3091349

[B33] Yamada I Brown BB Smith KR Zick CD Kowaleski-Jones L Fan JX Mixed land use and obesity: an empirical comparison of alternative land use measures and geographic scales Prof Geogr 2012 64 157 177 10.1080/00330124.2011.583592 22665941 PMC3365604

[B34] Rundle A Neckerman KM Freeman L Lovasi GS Purciel M Quinn J Richards C Sircar N Weiss C Neighborhood food environment and walkability predict obesity in New York City Environ Health Perspect 2009 117 442 447 19337520 10.1289/ehp.11590 PMC2661915

[B35] Larson N Story MT Nelsin MC Neighborhood environments: disparities in access to healthy foods in the U.S Am J Prev Med 2009 36 74 81 10.1016/j.amepre.2008.09.025 18977112

[B36] Oreskovic NM Winickoff JP Kuhlthau KA Romm D Perrin JM Obesity and the built environment among Massachusetts children Clin Pediatr (Phila) 2009 48 904 912 10.1177/0009922809336073 19487763

[B37] Bell JF Wilson JS Liu GC Neighborhood greenness and 2-year changes in body mass index of children and youth Am J Prev Med 2008 35 547 553 10.1016/j.amepre.2008.07.006 19000844 PMC2649717

[B38] Nielsen TS Hansen KB Do green areas affect health? Results from a Danish survey on the use of green areas and health indicators Health Place 2007 13 839 850 10.1016/j.healthplace.2007.02.001 17392016

[B39] Smith KR Brown BB Yamada I Kowaleski-Jones L Zick CD Fan JX Walkability and body mass index. Density, design, and new diversity measures Am J Prev Med 2008 35 237 244 10.1016/j.amepre.2008.05.028 18692736

[B40] Frank LD Andresen MA Schmid TL Obesity relationships with community design, physical activity, and time spent in cars Am J Prev Med 2004 27 87 96 10.1016/j.amepre.2004.04.011 15261894

[B41] Lopez-Zetina J Lee H Friis R The link between obesity and the built environment. Evidence from an ecological analysis of obesity and vehicle miles of travel in California Health Place 2006 12 656 664 10.1016/j.healthplace.2005.09.001 16253540

[B42] Pendola R Gen S BMI, auto use, and the urban environment in San Francisco Health Place 2007 13 551 556 10.1016/j.healthplace.2006.02.004 16621666

