# Supplementary figures and images for: Body mass index and overweight in relation to residence distance and population density: experience from the Northern Finland birth cohort 1966
Source: BMC Public Health. 2013 Oct 8;13:938. doi: 10.1186/1471-2458-13-938 (PMC3851578; doi:10.1186/1471-2458-13-938)

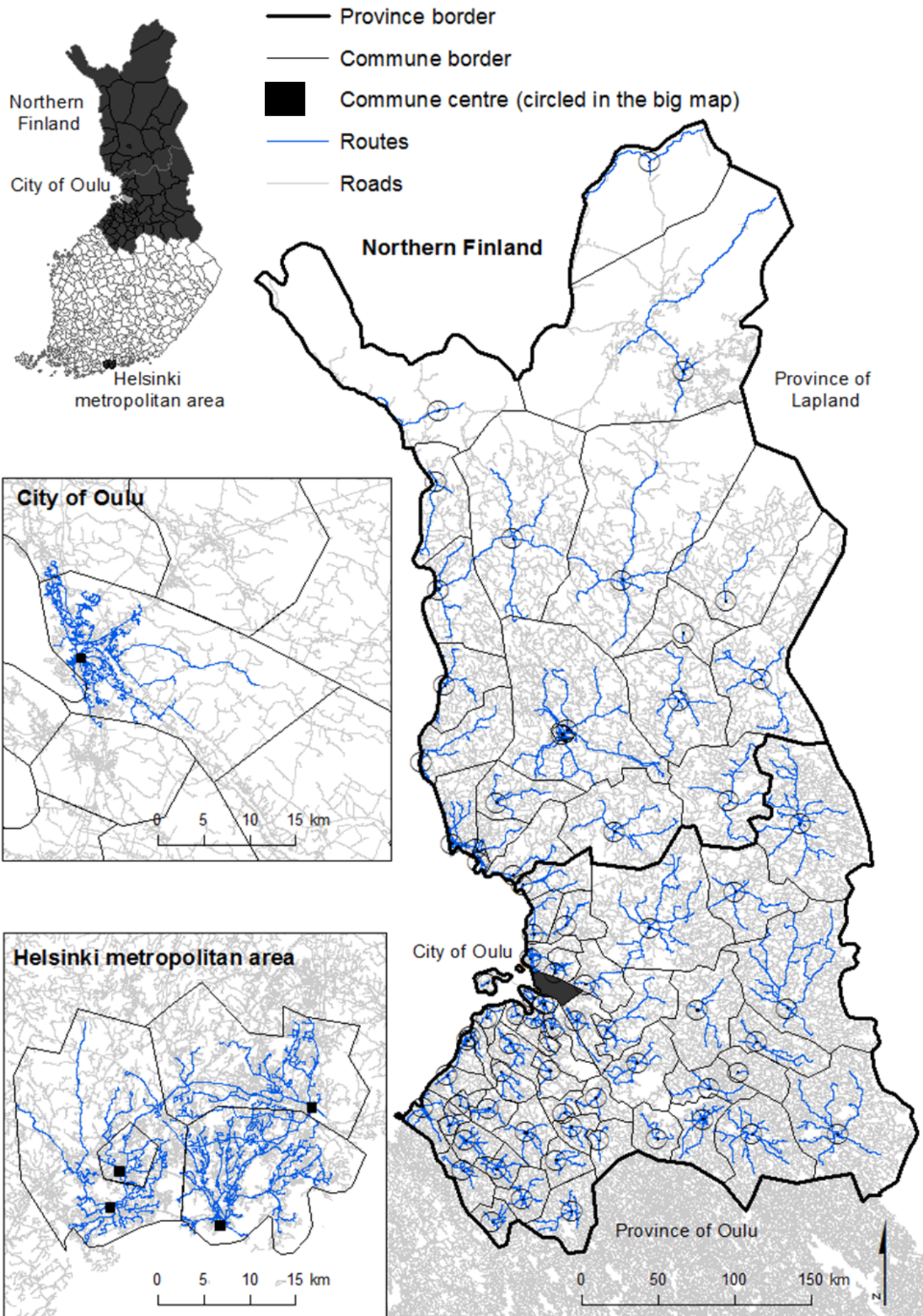

Supplement: Additional file 1: Figure S1 — Detailed map of the area studied. Detailed map of the area studied, showing the entire road network (marked in grey) and the roads used to calculate each individual’s travel distance to the resident commune’s centre (blue). [file 1471-2458-13-938-S1.pdf]

# MEN

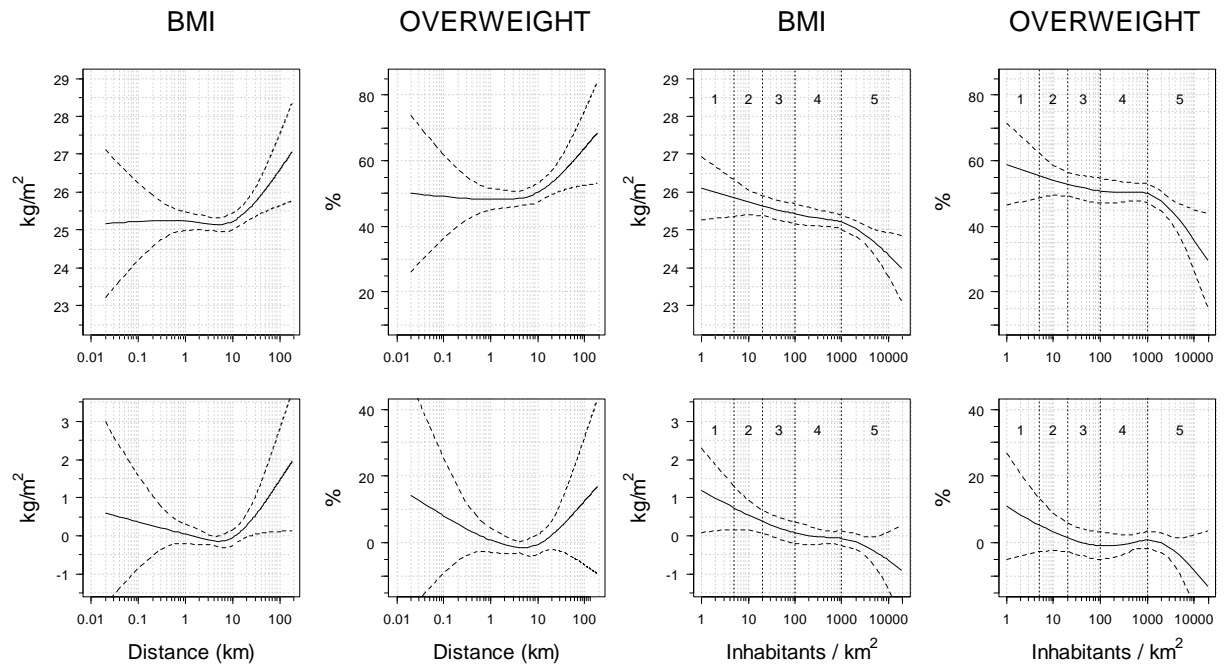

# WOMEN

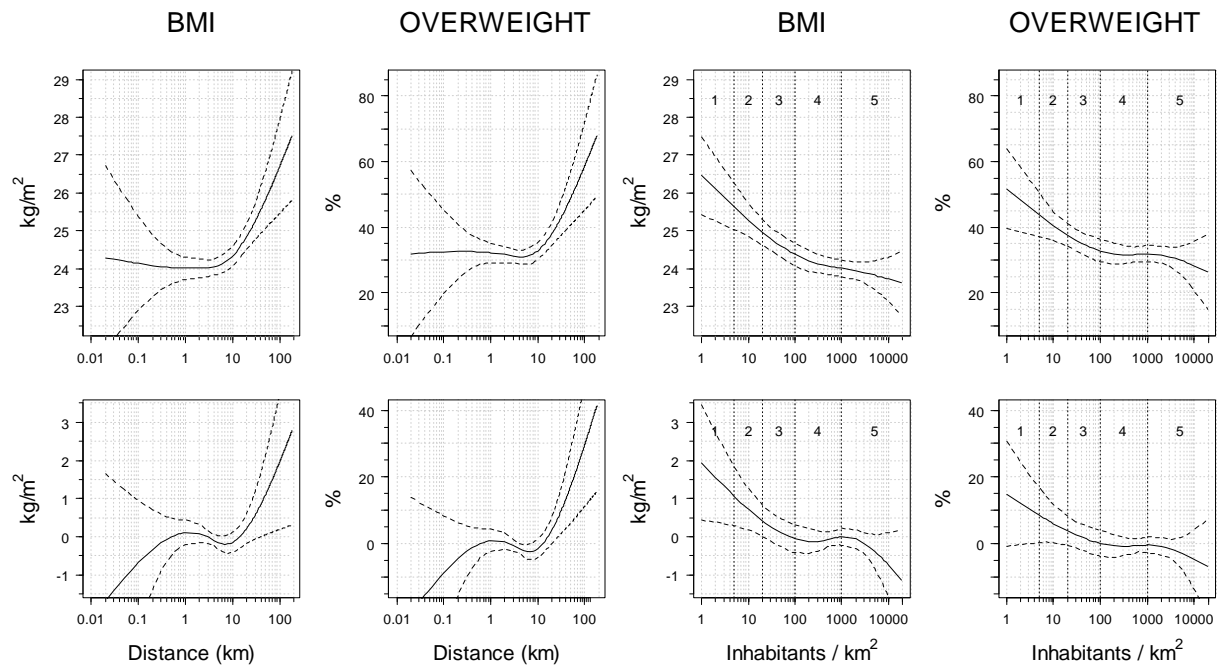

Supplement: Additional file 2: Figure S2 — BMI (kg/m2) and overweight (BMI ≥ 25 kg/m2) according to residence distance and population density, by sex. Body mass index (BMI; kg/m2) and percentage of overweight (BMI ≥ 25 kg/m2) in relation to individual’s road distance (km) to the resident commune’s densest grid, and on population density of the resident grid (inhabitants/km2), separately for men and women. Continuous line indicates the regression-based estimate for BMI and the prevalence of overweight, smoothed by a cubic spline with 4 degrees of freedom (95% confidence bands shown by dashed lines). Residential area types are marked by Arabic numerals: 1 scattered settlements; 2 rural areas proper; 3 transition zones; 4 built-up areas & suburbs; 5 high-rise centres. Upper rows: crude BMI and prevalence of overweight. Lower rows: regression-based gradients compared with the baseline, adjusted for marital status, occupational class, education, leisure-time and occupational physical activity, alcohol consumption and smoking. [file 1471-2458-13-938-S2.pdf]
